# Supplementary material for: Content-rich biological network constructed by mining PubMed abstracts
Source: BMC Bioinformatics. 2004 Oct 8;5:147. doi: 10.1186/1471-2105-5-147 (PMC528731; doi:10.1186/1471-2105-5-147)
Supplement: Additional File 5 — The original Chilibot query results of the term "long-term potentiation (LTP)" and 22 other terms, limiting the latest references analyzed to the years 1990, 1995, 2000, and 2004. [file 1471-2105-5-147-S5.bz2 › chilibotAdditionalFile5/ltp1995/html/AMPA_CAMKII.html]

 


 **AMPA** and **CAMKII** 
  
Found 8 abstracts in PubMed,  **8 abstracts were retrieved and analyzed**.  


---

 Search Google  |
 PDF files only 
|  EDU domain only 

---

**Interactive relationship** (e.g. stimulation, inhibition, etc)

- We conclude that local calcium influx through  **AMPA**  gated channels followed by activation of  **CaMkII**  is an important step for spinule retraction during dark adaptation.  Ref: 8528466 Eur J Neurosci, 1995
- We propose that entry of calcium into CA1 neurons activates calcium calmodulin dependent protein kinase II  [ **CAMKII** ] , which leads to an enhanced responsiveness of synaptic  **AMPA**  receptor channels.  Ref: 7917294 Neuron, 1994
- Here we report that in acutely isolated rat spinal dorsal horn DH neurons, the alpha amino 3 hydroxy 5 methyl 4 isoxazole propionic acid  **AMPA**  kainate and N methyl D aspartate NMDA receptors can be regulated by endogenous and exogenous calcium calmodulin dependent protein kinase II  [ **CAMKII** ]  CaM KII.  Ref: 7884477 J Neurophysiol, 1994

**Parallel relationship** (e.g. studied together, co-existance, homology, etc.)

- Phosphorylation of  **AMPA**  type glutamate receptors by calcium calmodulin dependent protein kinase II  [ **CAMKII** ]  and protein kinase C in cultured hippocampal neurons.  Ref: 7509863 J Neurosci, 1994
